# 

**Published:** 1892-06

**Authors:** 


### BRISTOL MEDICO-CHIRURGICAL JOURNAL

A Quarterly Journal of the Medical Sciences for the West of England and South Wales

*PUBLISHED UNDER THE AUSPICES OF*
*THE BRISTOL MEDICO-CHIRURGICAL SOCIETY.*

"Scire est nescire, nisi id me

Scire alius sciret."

*Editor:* R. SHINGLETON SMITH, M.D., B.Sc.
WITH WHOM ARE ASSOCIATED
BARCLAY J. BARON, M.B., C.M. F. R. CROSS, M.B.
J. MICHELL CLARKE, M.B., M.A. W. H. HARSANT.
*Assistant-Editor:* L. M. GRIFFITHS.

BRISTOL: J. W. ARROWSMITH;
London: J. & A. Churchill, 11 New Burlington Street.

*Price One Shilling and Sixpence.*

